# Cholecystoduodenal fistula resulting in gallstone ileus: A path paved by stone

**DOI:** 10.1002/ccr3.3943

**Published:** 2021-03-09

**Authors:** Brian T. Lee, Ahmad Mahamid, Jawad Ahmad, Parissa Tabrizian

**Affiliations:** ^1^ Recanati/Miller Transplantation Institute Division of Liver Diseases Department of Medicine Icahn School of Medicine at Mount Sinai New York NY USA; ^2^ Recanati/Miller Transplantation Institute Division of Abdominal Transplantation Department of Surgery Icahn School of Medicine at Mount Sinai New York NY USA

**Keywords:** cholecystoduodenal fistula, enterolithotomy, gallstone ileus

## Abstract

This report showcases the initial management of gallstone ileus which includes proper biliary assessment to assist with operative planning. While an uncommon condition, surgical management is crucial, although methodology may be variable.

## Graphical abstract

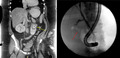

## 1 CASE

Gallstone ileus is defined by a partial or complete mechanical bowel obstruction due to gallstone impaction in the bowel lumen. Ensuing pressure necrosis from cholecystitis can result in the development of a cholecystoenteric fistula, leading to subsequent bowel obstruction. While uncommon, surgical management is crucial, although methodology may be variable.

**FIGURE 1:**
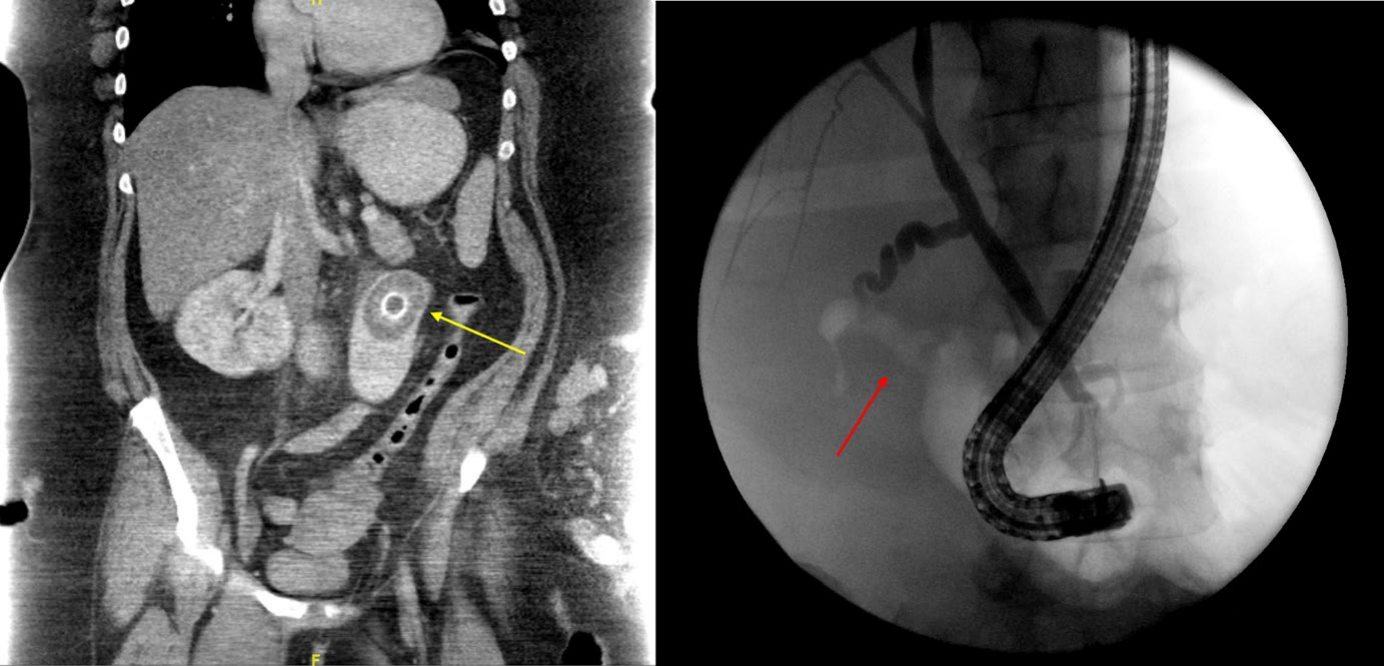
Left: Abdominal CT scan showing impacted gallstone in bowel lumen (yellow arrow). Right: Fluoroscopic image during ERCP showing cholecystoduodenal fistula (red arrow)

**FIGURE 2:**
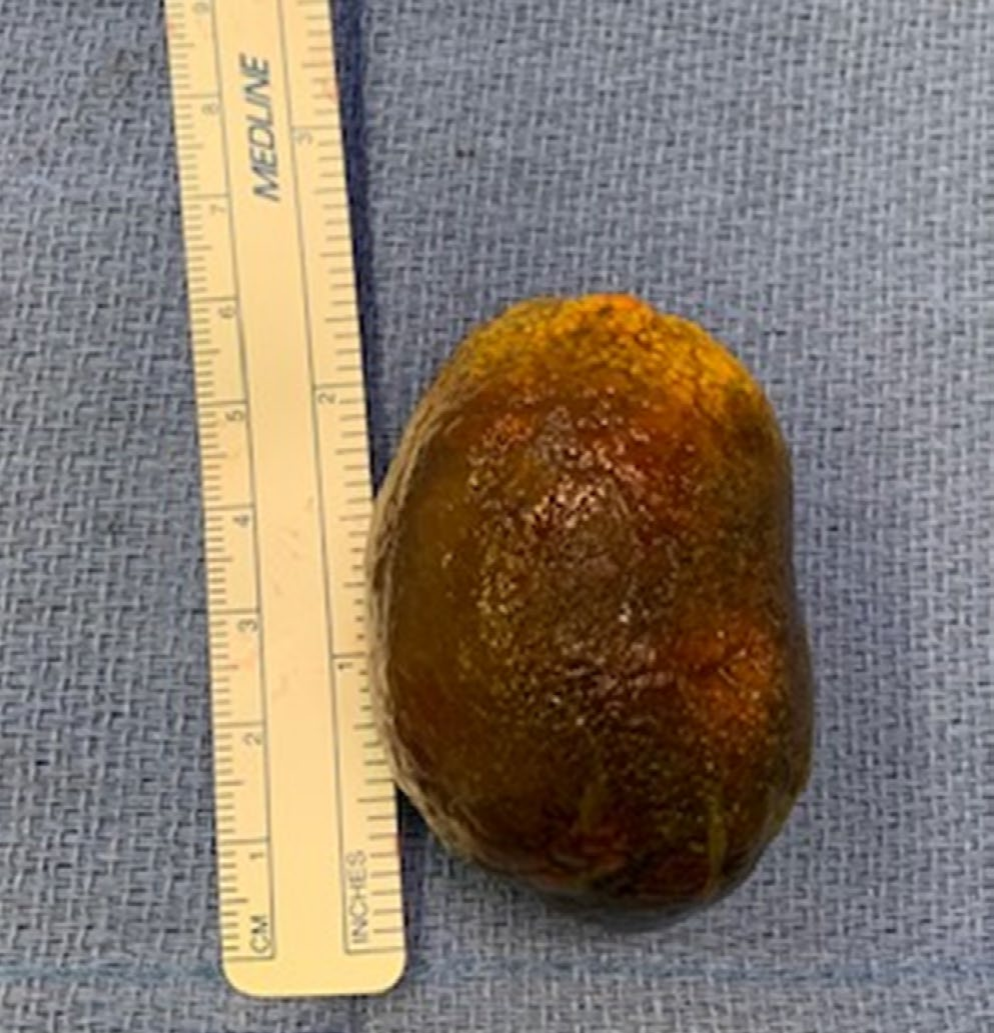
Extracted 6‐cm stone after enterotomy

A 35‐year‐old woman presented with abdominal pain and nausea for several days. She had a history of cholecystitis with an aborted cholecystectomy 1 year prior due to extensive inflammation. On this presentation, an abdominal CT scan showed air in the gallbladder fossa along with a dilated small bowel proximal to a transition point in the proximal jejunum with a large gallstone seen (Figure 1). Subsequently, an endoscopic retrograde cholangiopancreatophy was performed with ulceration seen in the duodenal bulb. A balloon occlusion cholangiogram revealed no filling defects in the biliary tree, but a tract of air connecting the gallbladder to the duodenal bulb, concerning for a cholecystoduodenal fistula (Figure 1). She underwent a laparotomy with an enterotomy to extract a 6‐cm stone (Figure 2). Due to the extensive adhesions, cholecystectomy was deferred. She had an uneventful postoperative course and was discharged home.

## 2 DISCUSSION

The initial surgical approach is enterolithotomy. Beyond this, surgical approaches for the management of the gallbladder and cholecystoenteric fistulas remain debated due to worse postoperative outcomes compared to enterolithotomy alone.^1^ Additionally, spontaneous closures of cholecystoenteric fistula have been reported, reducing the need for surgical intervention.^2^ In our case, surgical management of the gallbladder and cholecystoenteric fistula was deferred due to the lack of accessibility related to severe inflammation and adhesions.

## CONFLICT OF INTEREST

No financial support or conflicts of interest were identified for this study for all authors. No data accessibility available.

## AUTHOR CONTRIBUTIONS

BTL: involved in initial draft, critical revision of draft, and approved final draft submitted. AM: involved in critical revision of draft and approved final draft submitted. JA: involved in critical revision of draft and approved final draft submitted. PT: involved in critical revision of draft and approved final draft submitted.
